# Preventive and Therapeutic Effects of MG132 by Activating Nrf2-ARE Signaling Pathway on Oxidative Stress-Induced Cardiovascular and Renal Injury

**DOI:** 10.1155/2013/306073

**Published:** 2013-03-07

**Authors:** Wenpeng Cui, Yang Bai, Ping Luo, Lining Miao, Lu Cai

**Affiliations:** ^1^Department of Nephrology, The Second Hospital of Jilin University, 218 Ziqiang Street, Changchun, Jilin Province 130041, China; ^2^KCHRI at the Department of Pediatrics, University of Louisville, 570 South Preston Street, Baxter I, Suite 304F, Louisville, KY 40202, USA; ^3^Department of Cardiology, The People's Hospital of Jilin Province, Changchun 130021, China

## Abstract

So far, cardiovascular and renal diseases have brought us not only huge economic burden but also serious society problems. Since effective therapeutic strategies are still limited, to find new methods for the prevention or therapy of these diseases is important. Oxidative stress has been found to play a critical role in the initiation and progression of cardiovascular and renal diseases. In addition, activation of nuclear-factor-E2-related-factor-2- (Nrf2-) antioxidant-responsive element (ARE) signaling pathway protects cells and tissues from oxidative damage. As a proteasomal inhibitor, MG132 was reported to activate Nrf2 expression and function, which was accompanied with significant preventive and/or therapeutic effect on cardiovascular and renal diseases under most conditions; therefore, MG132 seems to be a potentially effective drug to be used in the prevention of oxidative damage. In this paper, we will summarize the information available regarding the effect of MG132 on oxidative stress-induced cardiovascular and renal damage, especially through Nrf2-ARE signaling pathway.

## 1. Introduction

The World Health Organization reports that chronic diseases as the leading cause of mortality in the world cause approximately 17 million people to die prematurely each year and keep steadily growing [1, 2]. What is more, this largely invisible epidemic is the worst in low- and middle-income countries, which could forego billions of dollars in national income as a result of these diseases. For example, the estimated losses in China from 2005 to 2015 are 558 billion dollars [1]. Cardiovascular diseases (CVD), a group of common chronic diseases, are the largest causes of morbidity and mortality worldwide. Chronic kidney disease (CKDs), also known as a microvascular disease, is an increasing public health concern too. CKD not only increases the risk of CVD and disease expenditure but also has a major impact on patients, health services, and society burden [3–5]. Thus, it is a priority to find effective drugs to treat CVD and CKD. 

Epidemiological studies have shown several risk factors for patients with CVD and CKD, such as heredity [6, 7], diabetes [8, 9], anemia [10], and hyperlipidemia [11, 12], but nontraditional risk factors such as oxidative stress may also contribute to these diseases [13, 14]. Our understanding of how oxidative stress contributes to cardiovascular and renal diseases has undergone considerable evolution over the past two decades. In recent years, reactive oxygen species (ROS) have come to be recognized as taking part not only in normal intracellular signaling for survival, but also in contributing to cytotoxicity [15]. Therefore, antioxidant therapy seems a preventive or therapeutic solution for the oxidative damage. Reportedly antioxidants such as vitamin E have been used in the treatment of human cardiovascular and renal disease; however, despite that there is one study supporting the therapeutic effect of vitamin E on these diseases [16], most of the clinical studies have failed to materially impact the course of the diseases [17, 18]. The possible reasons might include inefficiency of monoantioxidant used such as vitamin E only. Therefore, supplemental or upregulating endogenous multiple antioxidant levels may be a more efficient approach than mono-antioxidant therapy.

There are highly regulated cellular defense systems, including the redox-sensitive nuclear-factor-E2-related-factor-2- (Nrf2-) antioxidant-responsive element (ARE) pathway. Nrf2 is a transcription factor to regulate the expression of a battery of antioxidant genes and other cytoprotective phase II detoxifying enzymes through binding ARE [19, 20]. Therefore, Nrf2-ARE pathway promises to be a valuable therapeutic target for the prevention of oxidative stress and damage. Accumulating investigation has demonstrated that proteasome inhibitor MG132 could protect cells and tissues against oxidative damage because it could activate the Nrf2-ARE signaling pathway, leading to an upregulation of detoxifying and antioxidant genes [21–24]. In this paper, we thus focus on the antioxidant effect of MG132 on oxidative stress-induced cardiovascular and renal diseases.

## 2. Oxidative Stress and Nrf2-ARE Signaling Pathway

### 2.1. Oxidative Stress

ROS, a necessary evil of aerobic life, are routinely produced as a byproduct of aerobic metabolism, oxidative phosphorylation, environmental stressors, disease, or even natural aging process [25]. ROS generation is an important signaling mechanism in cells [26]. Our body is under constant oxidative attack from ROS so that a complex antioxidant system that generally defends this attack in balance has been evolved [15]. Oxidative stress is defined by the imbalance between the production of ROS and the endogenous antioxidant mechanisms that counteract the effects of ROS or repair the resulting damages [27]. Under physiological conditions, several tightly controlled oxidative pathways contribute towards ROS productions, while several endogenous antioxidant enzymatic mechanisms account for ROS depletion [28]. Either caused by reduced detoxification or increased generation, ROS can lead to widespread and indiscriminate cellular damage. As the central cause of oxidative stress, ROS at homeostatic levels have diverse actions on cell function. For instance, ROS can activate protein kinases (such as mitogen-activated protein kinases (MAPK)) [29] and upregulate redox-sensitive factors (such as NF *κ*B and AP-1) [30, 31]. On the other hand, it can be detrimental to cellular homeostasis by leading to opening ion channels [32] and major cellular macromolecules damage, including lipid peroxidation [33], DNA oxidation [34], and protein modification [35]. These damages, if left unrepaired, can lead to mutations that cause diseases.

### 2.2. Mechanism of the Nrf2-ARE Signaling Pathway in Oxidative Stress-Associated Injury

**Figure 1:**
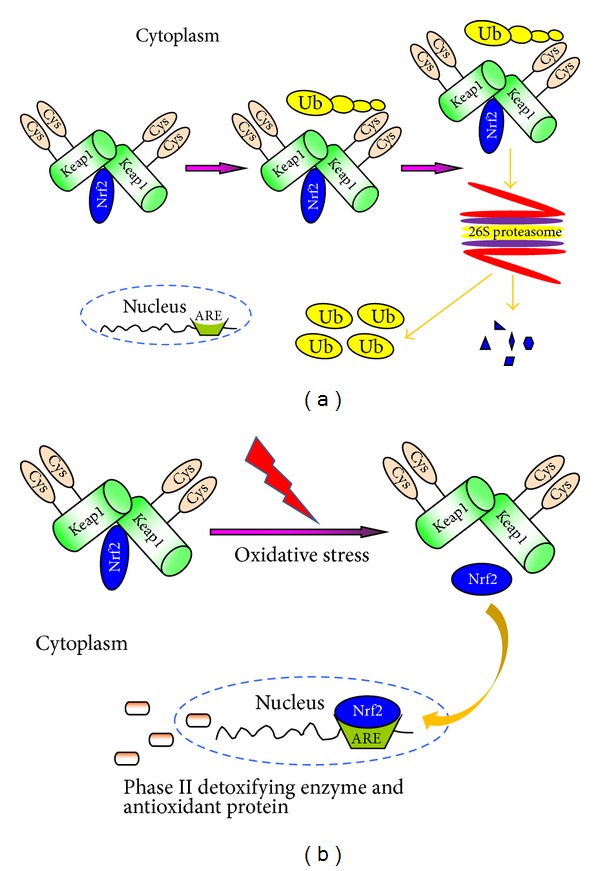
(a) Nrf2/Keap1-ARE signaling pathway under physical condition and (b) oxidative stress condition. ARE: antioxidant-responsive element; Cys: cysteine; Keap1: Kelch-like ECH-associated protein 1; Nrf2: E2-related factor 2; Ub: ubiquitin.

There is an upsurge of interest in Nrf2-ARE system because it plays a key role in the cell's response to oxidative stress [36–38]. Nrf2, a cap-n-collar family of nuclear basic leucine zipper transcription factors, is the central of this system and regulates cellular defenses against ROS. Nrf2-ARE signaling pathway is regulated by complex and poorly understood mechanisms. Kelch-like ECH-associated protein 1 (Keap1), known as an actin cytoskeleton-associated protein, binds very tightly to Nrf2 and anchors this transcription factor in the cytoplasm [39]. Keap1 also serves as a substrate adaptor for Cullin-3 (Cul3) that binds to ring-box 1 to form the E3 ubiquitin-ligase complex. The latter ultimately leads to ubiquitination and proteasomal degradation of Nrf2; thereby the ability of Nrf2 to induce phase II detoxification enzyme genes is repressed, as shown in Figure 1 [40–43]. 

When exposed to various stimuli such as oxidative stress, certain antioxidants, and chemopreventive agents, the Nrf2/Keap1 complex will be disrupted by modifying two (Cys273 and Cys288) of the 25 cysteine residues of Keap1 [44], allowing the cytoplasmic-to-nuclear translocation of Nrf2. In the nucleus, Nrf2 increases gene expression of phase II detoxifying and/or antioxidant enzymes such as glutathione S-transferase (GST), superoxide dismutase (SOD), catalase (CAT), glutathione peroxidase (GPx), NAD (P) H:quinine oxidoreductase 1 (NQO1), and heme oxygenase 1 (HO-1) [45, 46]. As shown in Figure 1(b), the transcriptional activation of these antioxidant enzymes is thought to be mediated by ARE or electrophile response element, which is found at the 5-flanking region of the phase II detoxification enzyme genes [47]. 

Modification of the Nrf2/Keap1 complex and Nrf2 nuclear translocation is important to Nrf2-ARE-pathway-dependent gene expression, and several signaling pathways are associated with these processes. For example, one component of these pathways is MAPKs. Both extracellular signal-regulated kinase (ERK) and p38MAPK have been found to induce Nrf2 translocation and HO-1 expression through diallyl sulfide in HepG2 cells [48]. In addition, protein kinase C (PKC) is also associated with Nrf2-dependent antioxidant enzyme expression. Huang et al. reported that PKC promotes Nrf2 phosphorylation at Ser-40, which yields the dissociation of Nrf2 from Keap1 in HepG2 cells. Data revealed that PKC-induced Nrf2 phosphorylation is critical to ARE-dependent antioxidant enzyme expression [49, 50]. Taken together, regulation of the upstream kinases involved, such as phosphatidylinositol 3-kinase (PI3 K), ERK, and PKC, provides a valuable tool for the investigation of Nrf2/Keap1 complex-controlled gene transcription [51].

## 3. Effects of Ubiquitin-Proteasome System (UPS) and MG132 on Nrf2-ARE Signaling Pathway

### 3.1. UPS

Proteins in eukaryotic cells are continually being synthesized and degraded. Two proteolytic systems, the lysosomal systems and UPS, are mainly responsible for this homeostasis. The lysosomal system is the principal mechanism for degrading proteins with long half-life and is the only system in cells for degrading organelles and large protein aggregates or inclusions [52]. The UPS pathway, as a highly specific extralysosomal system, plays a pivotal role in the degradation of misfolded and damaged proteins within the eukaryotic cells. Moreover, the UPS is also essential for selective degradation of short-lived and regulatory proteins involved in a wide variety of fundamental cellular processes, including cell cycle control [53], apoptosis [54], transcriptional regulation [55], proliferation [56], cell surface receptors expression [57], ion channels modulation [58], and Nrf2 degradation [59].

**Figure 2:**
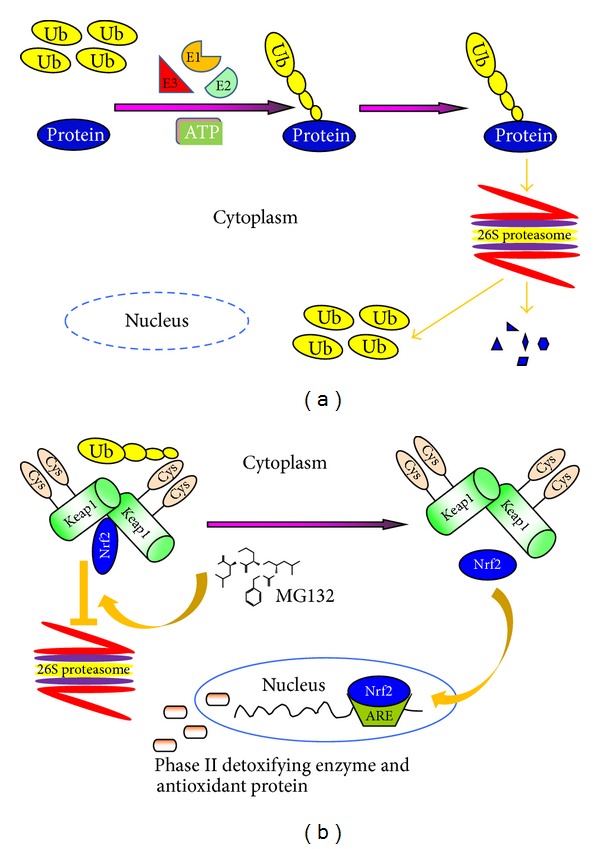
The process of target protein degeneration by UPS in eukaryotic cells (a) and mechanism of MG132 activate Nrf2/Keap1-ARE signaling pathway (b). Abbreviations: ARE: antioxidant-responsive element; Cys: cysteine; E1: ubiquitin-activating; E2: ubiquitin-conjugating; E3: ubiquitin-ligase; Keap1: Kelch-like ECH-associated protein 1; Nrf2: E2-related factor 2; Ub: ubiquitin.

The UPS consists of three parts: the 76-amino acid protein ubiquitin, the multisubunit complex 26S proteasome, and three enzymes, including ubiquitin-activating (E1), ubiquitin-conjugating (E2), and ubiquitin-ligase (E3) which are involved in a 3-step enzymatic cascade process [53, 60]. In an energy-dependent stepwise process catalyzed by three enzymes (E1, E2, and E3), target proteins for the proteasomal degradation are conjugated to multiple units of ubiquitin yielding a polyubiquitinated proteins. In the next step, unfolding ubiquitinated proteins are recognized, hydrolyzed, and then degraded by the 26S proteasome [61], which was illustrated in Figure 2(a). Proteasome, a highly conserved catalytic enzyme complex, is a large multisubunit protease and the most common form is known as 26S proteasome. It is composed of one catalytic 20S core particle (CP or 20S proteasome) and one or two 19S regulatory particles (RP or 19S proteasome) (Figure 2(b)). The 26S proteasome is a 2.5 MD protein complex which presents in the nucleus and cytoplasm of all eukaryotic cells [62, 63]. Known as 20S proteasome, the large core unit with a molecular mass of approximately 700 kDa is made up of two outer *α* rings and two inner *β* rings, which consists of 7 structurally similar *α* and *β* subunits, respectively [62]. The 20S proteasome contains proteolytic active sites that are sequestered within an interior space and performs several peptidolytic functions to maintain cellular homeostasis [64]. On the other hand, the 19S proteasome is able to recognize polyubiquitylated target proteins and take part in their deubiquitylating, unfolding, and translocation into the interior space of the 20S proteasome for destruction [62].

### 3.2. Proteasome Inhibitor MG132 and Nrf2-ARE Signaling Pathway

MG132 (Z-Leu-Leu-Leu-CHO), a peptide aldehyde proteasome inhibitor, was constructed by Roca et al. in 1994 and has been widely used in proteasome biology, allowing for the identification of new therapeutic targets and the development of novel therapeutic strategies. MG132 is a substrate analogue and potent transition-state inhibitor and mainly exhibits the chymotrypsin-like activity of the proteasome [65, 66]. When cells are exposed to this cell-permeable, potent, highly specific, and reversible proteasome inhibitor, MG132 will reduce degradation of ubiquitin-conjugated Nrf2 by inhibiting activity of the *β* subunits of the core particle of 26S proteasome without affecting its ATPase or isopeptidase activities. Subsequently, undegraded Nrf2 will be released from the Nrf2/Keap1 complex and translocate into the nucleus. Then Nrf2 binds to ARE and upregulates transcription of antioxidant genes (Figure 2(b)). 

The stabilization of Nrf2 by proteasome inhibition and subsequent transcriptional activation of its downstream genes have been shown in different cell types in earlier studies [24, 42, 67–69]. Recently, several studies have demonstrated that MG132 has the capacity of activating Nrf2-ARE signaling pathway in a variety of disease conditions [22, 70, 71]. This antioxidant response is known to be dose dependent. Low-dose MG132 exposure improves cellular fitness accompanied by the up-regulation of heat-shock proteins, GST, and Nrf2 [22, 68, 72] while high-dose MG132 yields an opposing effect that leads to apoptosis and even severe oxidative stress [73, 74]. Although the precise mechanism by which MG132 exerts antioxidant effects has not been fully understood, one well-accepted hypothesis is that the antioxidative effect of MG132 is related to the prevention of Nrf2 degradation through its suppression of UPS and subsequent translocation of Nrf2 from cytoplasm into the nucleus [41]. In Huang et al.'s study, the phosphorylation of Nrf2 at serine 40 appears to be a critical event in the release of Nrf2 from Keap1 and the translocation of Nrf2 from cytosol into the nucleus [49]. However, whether MG132 can provoke Nrf2 phosphorylation remains unknown; therefore, further investigations are needed to make this mechanism clear.

Despite that MG132 inhibition of proteasome results in an elevation of Nrf2 expression, the compensative induction of proteasome activity was also noticed. For instance, elevated proteasome subunit synthesis upon proteasome inhibition by MG132 is well conserved in human squamous cells [75, 76]. Interestingly, Nrf2, as a degradation target of proteasome, was also thought to mediate the proteasome recovery by increasing the 20S proteasome and the Pa28*αβ* (11S) proteasome regulator protein levels through a transcriptional feedback loop [77]. However, other studies demonstrated that the compensatory increase in proteasome subunit gene expression was Nrf1 dependent, instead of Nrf2 [75, 76]. Therefore, the exact mechanisms by which proteasomal activity is compensatively increased remain systemic studies.

## 4. Effect of MG132 on Oxidative Stress-Induced Cardiovascular and Renal Injury: Nrf2-Dependent Pathway

### 4.1. Preventive Effect of MG132

#### 4.1.1. Cardiovascular Injury

With regard to CVD, many of the pathogenic components of the disease are associated with oxidative stress, such as inflammation, LDL oxidation, and endothelial dysfunction. Overproduction and accumulation of ROS severely damage DNA, proteins, and lipids, resulting in further tissue damage and organ dysfunction. Compelling evidence supports the idea that supraphysiological levels of ROS (or called oxidative stress) play an important role in the pathophysiology of various CVDs, including endothelial dysfunction [78, 79], atherosclerosis [80, 81], and ischemia-reperfusion injury [82]. 

Our previous study indicated that high glucose could lead to ROS generation in both primary neonatal and adult cardiomyocytes from wild-type mouse heart. Whereas, in Nrf2 knockout cells from Nrf2 knockout mice, ROS were significantly higher under basal conditions and high glucose markedly further increased ROS production in concentration- and time-dependent manners [83]. Nrf2 was shown to mediate the basal expression and induction of ARE-controlled NQO1 and HO-1, at both mRNA and protein levels in cardiomyocytes [83]. Persuasive evidence has suggested that activation of antioxidant genes through Nrf2-ARE-dependent mechanism might yield protection against oxidative stress-associated injury in CVD [19, 84]. This antioxidant effect of proteasome inhibitor MG132 was confirmed by a Germany group [23]. Exposure to 0.5 *μ*M MG132 for 48 h proved to be nontoxic and protected neonatal rat cardiac myocytes against H_2_O_2_-mediated oxidative stress [23]. Another study from China investigated the effects of long-term MG132 treatment on cardiac hypertrophy *in vivo*. This study showed that treatment with MG132 (0.1 mg/kg/day) for 8 weeks attenuated pressure-overload-induced cardiac hypertrophy and improved cardiac function in abdominal aortic banding rats [85]. Recently a study from our group showed that therapeutic effect of MG132 on diabetic cardiomyopathy is associated with its suppression of proteasomal activities [86]. Mechanistically MG132 may upregulate Nrf2-mediated anti-oxidative function and downregulate NF-*κ*B-mediated inflammation.

In a similar study, we treated STZ-induced diabetic mice with sulforaphane at 0.5 mg/kg daily in five days of each week for 3 months. Sulforaphane treatment completely prevented diabetes-induced aortic pathogenic changes by attenuating oxidative stress, inflammation, and fibrosis in the aorta [87]. The aortic protection by sulforaphane treatment from diabetes was also accompanied with a significant up-regulation of Nrf2 expression and function (reflected by its downstream genes: HO-1, NQO1, and SOD1 expression) [87]. MG132 was also used in several vascular diseases. For instance, nontoxic inhibition of the proteasome using MG132 was found to protect against oxidative stress-induced endothelial dysfunction through increasing depressed SOD1 expression [71]. This finding is in line with a previous report that MG132 could liberate Nrf2 from Keap1 and translocate to nucleus to bind DNA with up-regulation of its downstream antioxidant genes [24]. Hemin is released from hemoglobin after central neuronal system hemorrhage and may cause ROS accumulation which contributes to cell loss in surrounding tissue. Pretreatment with 1 *μ*M MG132 for 2 h prevented approximately half of heme-mediated oxidative injury by up-regulation of Nrf2 and HO-1 [88].

#### 4.1.2. Renal Injury

Similar to CVD, oxidative stress is also the major player in the process of many kidney diseases, including acute kidney injury (AKI) [89, 90], ischemia reperfusion-induced renal injury [91], primary glomerulonephritis [92–96], diabetic nephropathy [97–101], lupus nephritis [102–104], and antineutrophil cytoplasmic antibodies-associated vasculitis [105, 106].

Previous work has indicated that impaired renal function in hypercholesterolemic pigs is improved by chronic proteasome inhibition with MLN-273 [107]. In a recent study, enhanced renal proteasome activity was found during lipopolysaccharide-induced AKI in human kidney cells. Suppression of proteasome activity using 10 *μ*M MG132 for 18 h can attenuate lipopolysaccharide-induced AKI [108]. In another AKI model, cisplatin-induced nephrotoxicity was markedly ameliorated by MG132 treatment both *in vivo* and *in vitro* [109].

Antifibrotic effect of MG132 at low doses has been observed in rat renal fibroblasts and mesangial cells [110, 111]. As we know, oxidative stress plays an important role in pathogenesis of diabetic nephropathy. Zheng et al. provided experimental evidence indicating that Nrf2-ARE signaling pathway activation by sulforaphane or cinnamic aldehyde can be used therapeutically to relieve renal damage induced by type 1 diabetes. This idea was confirmed by our recent study [112]. We treated type 1 diabetic mice with sulforaphane at 0.5 mg/kg daily for five days for each for 3 months. At the end of 3-month treatment with sulforaphane one set of mice was sacrificed to perform the experimental measurements (3-month time point). The second set of mice was aged for 3 additional months without further sulforaphane treatment (6 month time point). Our results revealed that sulforaphane significantly prevented diabetes-induced renal inflammation, oxidative damage, and fibrosis by activation of Nrf2-ARE signaling pathway in the kidney at 3-month time point, but not at 6-month time point, suggesting the requirement of continual use of sulforaphane for its sustained effect [112]. In another STZ-induced diabetes rat model, MG132 was administered at a dose of 10 *μ*g/kg/day via intraperitoneal injection once daily for 3 months. After MG132 treatment, renal Nrf2 and its downstream antioxidants (SOD1, CAT, and GPx) were upregulated and diabetic renal damage was also improved [22].

### 4.2. Therapeutic Effect of MG132

#### 4.2.1. Cardiovascular Injury

A recent study from our group suggested that therapeutic effect of MG132 on diabetic cardiomyopathy is associated with its suppression of proteasomal activities [86]. Diabetic mice showed significant cardiac dysfunction, heart structural derangement, and remodeling (fibrosis and hypertrophy), as well as increased systemic and cardiac oxidative damage and inflammation. All of these pathogenic changes were reversed by MG132 treatment. In addition, MG132 treatment significantly increased cardiac expression of Nrf2 and its downstream antioxidant genes and also significantly decreased the expression of I*κ*-B and the nuclear accumulation and DNA binding activity of NF-*κ*B in the heart. Therefore, the possible mechanisms might include both up-regulating Nrf2-mediated anti-oxidative function and downregulating NF-*κ*B-mediated inflammation induced by MG132.

#### 4.2.2. Renal Injury

The therapeutic effect of MG132 on diabetic nephropathy was also reported by our group [113]. Three-month old transgenic type 1 diabetic (OVE26) mice displayed renal dysfunction with albuminuria and then were treated with MG132 (10 *μ*g/kg/day). After 3-month treatment with MG132, diabetes-induced renal oxidative damage, inflammation, fibrosis, and eventual dysfunction were significantly attenuated accompanied with a significant decrease in 20S proteasome activity decrease and activation of Nrf2-ARE signaling pathway. *In vitro* study using human renal tubular HK11 cells confirmed the role of Nrf2 in the prevention of diabetes-induced renal damage. HK11 cells were treated with high glucose (27.5 mM) for 48 h. During that time, MG132 (2 *μ*M) and palmitate (300 *μ*M) were added in the last 9 h and 6 h, respectively. Immunofluorescent staining for Nrf2 showed that Nrf2 expression and nuclear accumulation were decreased in high glucose plus palmitate group but increased in MG132 treatment group. MG132 treatment also significantly prevented the increase of connective tissue growth factor overexpression in the cells treated with high glucose plus palmitate. What's more, silencing the Nrf2 gene with its specific siRNA abolished MG132 decrease of high glucose and palmitate-induced connective tissue growth factor overexpression. These results suggested that MG132 upregulates Nrf2 function via inhibition of diabetes-increased proteasomal activity, leading to the therapeutic effect on diabetic nephropathy.

### 4.3. Dose-Dependent Effects of MG132 on Cardiovascular and Renal Injury

It should be mentioned that whether cells have beneficial response to MG132 also depend on several factors, including the type of cells, the dose of MG132, and the exposure time. Contrast to the studies discussed above, several studies in cardiac myocytes showed an opposite conclusion. Exposure of myocytes to high doses of MG132 (10 *μ*M) in short term enhanced the cellular damage [114, 115]. Available evidence suggests that toxic inhibition of proteasome function induces programmed cell death in proliferating endothelial cells [116]. Similarity, proteasome inhibitor MG132 has been shown to affect cell growth and death through formation of ROS and depletion of GSH in As4.1 juxtaglomerular cells [117–119]. In order to explain this interesting phenomenon, Meiners et al. have systemically analyzed dose-dependent effects of proteasome inhibition with MG132 using human umbilical cord vein cells [120]. They found that nontoxic doses of MG132 (70 nM) induced a defined, dose-dependent transcriptional response by up-regulating anti-oxidative enzymes (e.g., SOD1, GPx) that were accompanied by protection against H_2_O_2_-induced oxidative stress, whereas high doses of MG132 (200 nM) induced apoptosis in endothelial cells [120]. In general, nontoxic proteasome inhibition might offer a new therapeutic approach for the treatment of oxidative stress-associated cardiovascular and renal diseases. 

## 5. Other Mechanisms by Which MG132 Protects Cells against Oxidative Damage

Although MG132 protects cardiovascular and renal damage from oxidative stress predominantly via Nrf2-ARE signaling pathway, other possible mechanisms should not be ignored. Among these mechanisms, the relatively well-studied one is I*κ*B-NF-*κ*B pathway. Recent studies suggested that hyperglycemia enhances 26S proteasome activity through peroxynitrite/superoxide-mediated PA700-dependent proteasomal activation, which elevates NF-*κ*B-mediated renal and aortic inflammatory response in early diabetes. Importantly, these alterations were abolished by MG132 administration [121]. Another *in vivo* study demonstrated that MG132 attenuated oxidative stress-induced damage by suppressing NF-*κ*B in coronary arterioles in type 2 diabetic mice, because increased NAD(P)H oxidase and NF-*κ*B activity in diabetes was attenuated by MG132 administration [122]. Similar situation was also found in H_2_O_2_-treated microvascular endothelial cells *in vitro* [123] and heart of rats with pressure overload *in vivo* [124]. Besides I *κ*B-NF-*κ*B pathway, MG132 can play a key role in cellular defense system by suppressing MAPK signaling pathway [125, 126] and blocking the degradation of vascular protective molecules [127].

## 6. Conclusions

Accumulating observation has illustrated that a great range of cardiovascular and renal diseases have been associated with oxidative stress. Given that Nrf2-ARE signaling pathway plays critical roles in preventing oxidative stress-associated injury, Nrf2 activators are supposed to be used clinically as a new strategy. In a phase 2, double-blind, randomized, placebo-controlled clinic trial, Dinkova-Kostova et al. used bardoxolone methyl, which has the ability to activate Nrf2 [128], to treat 227 patients with CKD for 52 weeks [129]. Results suggested that patients receiving bardoxolone methyl had significant increases in estimated glomerular filter rate compared with those given placebo, accompanied by only mild adverse effects, such as muscle spasms, hypomagnesemia, and gastrointestinal effects. Similar outcomes were obtained in a subgroup study for diabetic nephropathy [129]. With the recent US Food and Drug Administration approval of bortezomib (Velcade1) for the treatment of relapsed multiple myeloma, the proteasome inhibition has been established as a powerful and promising therapeutic strategy for oxidative stress damage [130, 131]. Although, to our knowledge, no evidence has been proved that MG132 can be used in patients with oxidative stress-induced cardiovascular and kidney diseases, it is increasingly apparent that MG132 has the antioxidant effect by up-regulation of Nrf2-ARE signaling pathway both *in vitro* and *in vivo*. Thus, MG132 may become another candidate for clinical application for the patients with cardiovascular and renal diseases. However, what is the dose window of MG132 in treatment of oxidative damage in human disease? What is the mechanism of MG132 to promote Nrf2 to release from Keap1? All these questions remain unanswered yet. Therefore, further research focusing on the effect of MG132 on Nrf2-ARE signaling pathway and the underlying mechanisms is urgently needed.
